# The DREB2C.L-IAGLU module contributes to long-term heat stress via sugar metabolism in cucumber

**DOI:** 10.1093/hr/uhaf341

**Published:** 2025-12-11

**Authors:** Xiao Ma, Chuang Li, Yong Yuan, Xitong Zhong, Yafei Huang, Jiacai Chen, Yan Geng, Yuyan Li, Zhaoyang Zhou, Ming Xin, Xiaolan Zhang, Jianyu Zhao

**Affiliations:** Sanya Institute of China Agricultural University, Sanya 572025, China; Beijing Key Laboratory of Growth and Developmental Regulation for Protected Vegetable Crops, Department of Vegetable Sciences, China Agricultural University, Beijing 100193, China; Sanya Institute of China Agricultural University, Sanya 572025, China; Beijing Key Laboratory of Growth and Developmental Regulation for Protected Vegetable Crops, Department of Vegetable Sciences, China Agricultural University, Beijing 100193, China; Sanya Institute of China Agricultural University, Sanya 572025, China; Beijing Key Laboratory of Growth and Developmental Regulation for Protected Vegetable Crops, Department of Vegetable Sciences, China Agricultural University, Beijing 100193, China; Sanya Institute of China Agricultural University, Sanya 572025, China; Beijing Key Laboratory of Growth and Developmental Regulation for Protected Vegetable Crops, Department of Vegetable Sciences, China Agricultural University, Beijing 100193, China; Beijing Key Laboratory of Growth and Developmental Regulation for Protected Vegetable Crops, Department of Vegetable Sciences, China Agricultural University, Beijing 100193, China; Beijing Key Laboratory of Growth and Developmental Regulation for Protected Vegetable Crops, Department of Vegetable Sciences, China Agricultural University, Beijing 100193, China; Sanya Institute of China Agricultural University, Sanya 572025, China; Beijing Key Laboratory of Growth and Developmental Regulation for Protected Vegetable Crops, Department of Vegetable Sciences, China Agricultural University, Beijing 100193, China; Sanya Institute of China Agricultural University, Sanya 572025, China; Beijing Key Laboratory of Growth and Developmental Regulation for Protected Vegetable Crops, Department of Vegetable Sciences, China Agricultural University, Beijing 100193, China; Beijing Key Laboratory of Growth and Developmental Regulation for Protected Vegetable Crops, Department of Vegetable Sciences, China Agricultural University, Beijing 100193, China; College of Horticulture and Landscape Architecture, Key Laboratory of Biology and Genetic Improvement of Horticultural Crops (Northeast Region), Northeast Agricultural University, Harbin 150030, China; Sanya Institute of China Agricultural University, Sanya 572025, China; Beijing Key Laboratory of Growth and Developmental Regulation for Protected Vegetable Crops, Department of Vegetable Sciences, China Agricultural University, Beijing 100193, China; Sanya Institute of China Agricultural University, Sanya 572025, China; Beijing Key Laboratory of Growth and Developmental Regulation for Protected Vegetable Crops, Department of Vegetable Sciences, China Agricultural University, Beijing 100193, China

## Abstract

Cucumber is an important vegetable crop with thermophilic but heat-sensitive growth characteristics. Heat stress threatens cucumber growth and development, leading to a decline in both quality and yield. However, the evaluation system and molecular mechanism of long-term heat tolerance remain unclear. Here, an evaluation system in response to long-term heat stress was established, and chlorophyll *a* content and catalase (CAT) activity were identified as key evaluation indices for determining the heat tolerance of cucumber seedlings. Transcriptomic and physiological analyses revealed that sugar metabolism played a pivotal role in the heat response. Notably, the expression of *CsIAGLU* (Indoleacetic Acid glucosyltransferase) was significantly upregulated in heat-tolerant genotype PS76, whereas it was not induced in the heat-sensitive genotype PWRG. Loss of function of *CsIAGLU* by gene editing resulted in increased sensitivity to heat stress along with higher sugar contents, accelerated stomatal closure, and chlorophyll degradation. Furthermore, CsDREB2C.L, a positive regulator of heat stress response, directly bound to the *CsIAGLU* promoter to enhance its expression. Overexpression of *CsDREB2C.L* and *CsIAGLU* maintained stable sugar contents, thereby keeping stomatal opening and sustaining leaf greening to resist heat stress. Taken together, our findings provide valuable insights into the mechanism of heat resistance in cucumber.

## Introduction

Due to the sessile lifestyle, plants are vulnerable to ambient temperature, which influences their seasonal growth and geographical distribution [1]. Global warming caused by human activity negatively affects the metabolism, growth, and development of plants, including seed germination, photosynthetic capacity, and pollen viability [2]. In recent years, heat stress has been reported as a significant factor to cause severe reduction in crop production [3, 4]. Therefore, exploring the mechanisms of plant response to heat stress is of great significance to cope with climate change and ensure agricultural production.

Plants activate an array of physiological and molecular responses under heat stress, including chlorophyll breakdown, reactive oxygen species (ROS) accumulation, stomatal opening, and transcriptional signal activation [2, 5, 6]. Multiple physiological parameters were used to construct the heat tolerance evaluation model to quickly evaluate plant heat tolerance, such as chlorophyll content, electrochemical conductivity, and antioxidant system [7–9]. Heat stress accelerates leaf senescence through chlorophyll breakdown, protein degradation, and expression of senescence-associated genes and chlorophyll catabolic genes [10–12]. In response to heat stress, plants open their stomata to cool the leaves through transpiration [13]. Furthermore, heat stress triggers the accumulation of misfolded proteins, which are bound by heat shock proteins (HSPs). This interaction releases heat shock factors (HSFs), enabling them to regulate downstream targets involved in heat stress responses [14]. In addition to HSFs, other transcription factors (TFs) and molecular members also participate in heat stress response, such as dehydration-responsive element binding (DREB) and UDP-glycosyltransferase (UGT) [15–17]. In tomato (*Solanum lycopersicum*), the pollen vigor and pollen germination were reduced in *SlhsfA1a* mutants, while they were increased in *SlHsfA1a*-OE plants under heat stress [18]. *Arabidopsis* AtDREB2A could be SUMOylated to enhance the stability under heat stress, also AtDREB2A activated the promoter of *AtHsfA3* to regulate the heat stress response [19, 20]. Furthermore, a rice quantitative trait locus (QTL), *Grain Size and Abiotic stress tolerance* 1 (GSA1) has been identified, which encodes a UGT gene (*OsUGT83A1*), and overexpression of *GSA1* enhanced the tolerance to salt and heat stresses [15].

Cucumber (*Cucumis sativus* L.) is an economically important vegetable crop throughout the world, which is popular owing to its fresh taste and rich nutrients. Although cucumber originates from the tropics, it is sensitive to heat stress, and the suitable growth temperature is 18°C–30°C [21]. In China, during summer, the temperature of cucumber cultivation often exceeds 35°C, leading to excessive hypocotyl growth, leaf sunburn, and a reduction of transplant survival rate. Heat stress will hinder photosynthesis, affect the transport of water and nutrients, and inhibit the growth and development of cucumber plants, ultimately resulting in the decline of yield and quality [22, 23]. Several heat stress-related QTLs have been identified on chromosome 1 and 3 of cucumber [21, 24]. Through homologous analysis, CsHSFs and CsHSPs have been identified in cucumber, nevertheless, their role in heat stress awaits further characterization [25]. *CsSPATULA* (*CsSPT*) and Abscisic acid-insensitive 5 (*CsABI5*) were found to participate in heat stress response in cucumber [11, 26]. The *Csspt* mutant plants displayed severe thermosensitive symptoms such as wilted leaves with brown margins and reduced root density under short-term heat stress (3 days) [26]. Transient silencing of *CsABI5* resulted in decreased heat-induced chlorophyll degradation and lower expression level of chlorophyll catabolic genes *CsPPH* (pheophytinase) and *CsPAO* (pheophorbide a oxygenase) [11]. However, the evaluation system and the molecular mechanism of long-term heat tolerance remain largely unknown in cucumber.

In this study, we established an evaluation system to assess cucumber seedlings under long-term heat stress (from seed germination to the seedling stage). To uncover the mechanism of heat tolerance in cucumber, transcriptomic analysis of XTMC (Northern China-type) revealed that sugar metabolism played a central role in response to heat stress. Consistently, sugar contents were higher in the heat-sensitive genotype than in the heat-tolerant genotype, suggesting that the accumulation of sugar may promote the closure of stomata and influence gas exchange. Furthermore, exogenous sugar treatment of the heat-tolerant genotype PS76 influenced heat resistance by regulating stomatal movement. Based on the transcriptomic analysis, we focused on a UGT gene, *CsIAGLU*, which was upregulated in the heat-tolerant genotype but not induced in the heat-sensitive genotype. This gene has been reported to negatively regulate the leaf petiole angle via modulating the endogenous Indoleacetic Acid (IAA) level in cucumber [27]. Functional analysis by the CRISPR-Cas9 system showed that *CsIAGLU* acts as a positive regulator of heat resistance by modulating sugar contents, chlorophyll contents, ROS system, and stomatal movement. Furthermore, we found that the DREB TF CsDREB2C encoded two transcripts: a canonical *CsDREB2C.L* and a short *CsDREB2C.S*, and the expression of *CsIAGLU* was specifically activated by CsDREB2C.L. Also, *CsDREB2C.L* positively regulated heat stress resistance via modulating sugar metabolism. Collectively, our study reveals the function of CsDREB2C.L-CsIAGLU module and the contribution of sugar in heat resistance in cucumber.

## Results

### Evaluation system of long-term heat tolerance at cucumber seedling stage

**Figure 1:**
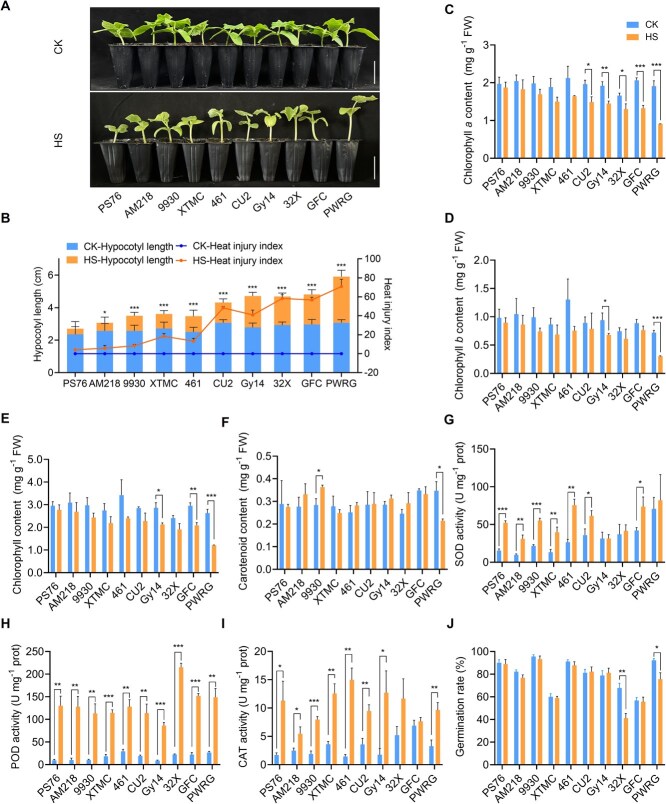
Phenotypic characterization of 10 cucumber genotypes under CK and HS conditions. (A) Representative phenotypes of 10 cucumber genotypes under CK and HS conditions. Scale bars, 5 cm. (B) The hypocotyl length and heat injury index. Asterisks represent significant differences in hypocotyl length. (C) Chlorophyll *a* content. (D) Chlorophyll *b* content. (E) Total chlorophyll content. (F) Carotenoid content. (G) SOD activity. (H) POD activity. (I) CAT activity. (J) Germination rate. Asterisks represent significant differences determined by Tukey’s test. ^*^P < 0.05; ^**^P < 0.01; ^***^P < 0.001.

To systematically dissect the phenotypic changes of cucumber in response to long-term heat stress, a total of 10 cucumber genotypes including four Southern China-type cucumbers (CU2, 32X, GFC, and PWRG), three Northern China-type genotypes (9930, XTMC, and 461), and three America cucumbers (PS76, AM218, and Gy14) were selected for heat treatment. Seeds of 10 cucumber genotypes were germinated in soil and subsequently transplanted to growth conditions of either control (CK) or heat stress (HS) treatment for 15 days. Under CK condition, the hypocotyl length (HL) showed no significant difference among the 10 genotypes (Fig. 1A and B). Under HS condition, some genotypes, such as PS76 and AM218, maintained green leaves and similar HLs as CK, while most genotypes displayed wilted and yellowed phenotypes with significantly elongated hypocotyls, in which the PWRG displayed the longest hypocotyl under HS condition. The heat injury index (HII) was measured to evaluate the sensitivity of different genotypes to HS. The results showed that PS76 and AM218 had strong heat resistance, while PWRG exhibited high sensitivity to HS (Fig. 1B).

Photosynthesis is an important biological process in plants, which is hypersensitive to HS [5]. To determine whether the photosynthesis system was influenced by HS, the contents of chlorophyll *a* (Chl *a*), chlorophyll *b* (Chl *b*), total chlorophyll (Chl), and carotenoid (Car) were measured under CK and HS conditions (Fig. 1C–F). Under HS condition, Chl *a*, Chl *b*, and Chl contents were significantly decreased in many genotypes, such as Gy14, GFC, and PWRG (Fig. 1C–E). As the major ROS scavengers, superoxide dismutase (SOD), peroxidase (POD), and catalase (CAT) activities were detected under CK and HS conditions. When seedlings were exposed to HS condition, the activities of SOD, POD, and CAT increased significantly in most genotypes (Fig. 1G–I). Moreover, the germination rates (GRs) of all genotypes were examined under 26°C and 42°C for 2 days. Under HS condition, 32X and PWRG showed a lower germination rate compared with other genotypes, suggesting they were more sensitive to HS (Fig. 1J).

To evaluate the heat tolerance of cucumber seedlings after 15-day heat treatment, we performed principal component analysis (PCA) on the morphological and physiological data of 10 cucumber genotypes, which could overcome the error of single index and the overlapping of each index. Three principal components (PC_1_, PC_2_, and PC_3_) can explain 83.543% of the total variance (54.617%, 17.854%, and 11.072%, respectively), which could represent the majority of the information contained original indices (Fig. S1A and B, Table S1). Then, the comprehensive index and membership function value were calculated to assess the heat tolerance value (H) of 10 cucumber genotypes (Table S2). To verify the reliability of the heat tolerance value, correlation analysis was performed between H and HII, and the results showed a significantly negative correlation between them, indicating that H can serve as a representative index in the evaluation system of long-term heat tolerance (Fig. S1C). To obtain the major index in response to HS, stepwise regression analysis was conducted with H as the dependent variable, and physiological indices as the independent variables. The model can be standardized into the following equation: H = 1.281*Chl *a* + 0.018*CAT-0.483 (*R*^2^ = 0.964, *F* = 120.450, *P* = 4 × 10^−6^). We found that Chl *a* and CAT could serve as major indices of long-term heat tolerance in cucumber seedlings. Additionally, 10 genotypes were clustered into three subgroups according to the heat tolerance values. PS76, AM218, 9930, and 461 were clustered into Group I, which was strongly resistant to HS. XTMC, CU2, 32X, and GFC were classified in the same group and had a moderate level of resistance, whereas PWRG was most sensitive to HS, belonging to Group III (Fig. S1D).

### Transcriptomic analysis of cucumber seedlings under HS

To obtain a deeper insight into the molecular mechanism of heat tolerance in cucumber, the well-studied cucumber genotype XTMC seedlings were sampled after 15 days of cultivation under CK and HS conditions, and RNA-seq analysis was performed with three biological replicates. A total of 4767 differentially expressed genes (DEGs) were identified between HS-XTMC and CK-XTMC, including 2710 upregulated genes and 2057 downregulated genes (Fig. S2A, Table S3). In a previous study, HSP20 members were reported to be induced under HS, such as *CsHSP15.9* (*CsaV3_4G025700*) and *CsHSP17.8* (*CsaV3_5G008780*) [28]. Here, we found that *CsHSP15.9* and *CsHSP17.8* were respectively induced 16.21- and 135.25-fold, indicating the high reliability of the transcriptomic analysis. To identify the underlying function of DEGs, Kyoto Encyclopedia of Genes and Genomes (KEGG) enrichment analysis showed that DEGs were mainly related to biosynthesis of secondary metabolites, starch and sucrose metabolism, and phenylpropanoid biosynthesis in metabolism (Fig. S2B, Table S4). To better understand the potential functions of DEGs in response to HS, 4767 DEGs were divided into three categories as biological process, cellular component, and molecular function by Gene Ontology (GO) enrichment analysis (Fig. S2C, Table S5). The results showed that the top 20 significant enrichment terms of three categories (*P*-value < 0.05). In the biological process, the main GO terms were ‘cellular carbohydrate metabolic process’, ‘response to salt stress’, and ‘response to acid chemical’. Some primary GO terms were identified in molecular function, such as ‘tetrapyrrole binding’, ‘oxidoreductase activity’, and ‘UDP-glycosyltransferase activity’. Based on the KEGG and GO enrichment analysis, we supposed that sugar metabolism might play an important role in heat resistance in cucumber. Hence, we focused on the expression patterns of genes related to starch and sucrose metabolism and UDP-glycosyltransferase activity (Fig. S2D, Table S6). Notably, we found a UGT gene *CsIAGLU* (Indoleacetic Acid glucosyltransferase) that was upregulated under HS condition. In our previous observation, *Csiaglu* mutants were more sensitive to environmental stress. To explore the possible function of *CsIAGLU* in response to HS, we detected the expression of *CsIAGLU* in three cucumber genotypes with different levels of heat tolerance (PS76 with strong heat resistance, XTMC with moderate heat resistance, and PWRG with high heat sensitivity) under CK and HS conditions. The results showed that *CsIAGLU* was sharply accumulated in heat-tolerant genotype PS76 and significantly upregulated in XTMC, while it was not induced in heat-sensitive cucumber PWRG under HS condition (Fig. S2E).

### Analysis of sugar metabolism in different genotypes under HS

**Figure 2:**
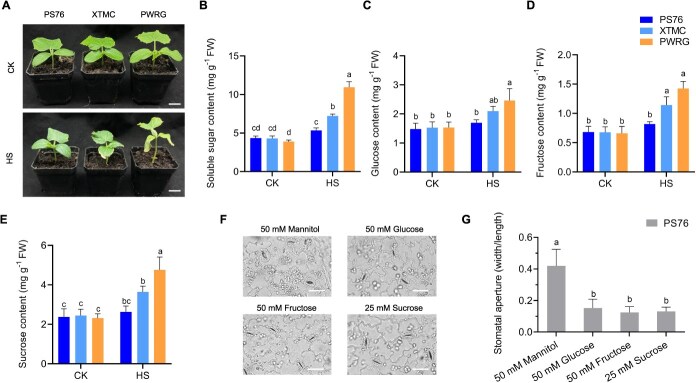
Analysis of sugar metabolism in PS76, XTMC, and PWRG. (A) Phenotypes of PS76, XTMC, and PWRG under CK and HS conditions. Scale bars, 2 cm. (B) Soluble sugar content. (C) Glucose content. (D) Fructose content. (E) Sucrose content. (F, G) Stomatal response to sugars in PS76 under treatment with 50 mM mannitol (osmotic control), 50 mM glucose, 50 mM fructose, and 25 mM sucrose. Scale bars, 25 μm. Letters represent significant differences according to Tukey’s test (*P* < 0.05).

To clarify whether HS affected the sugar contents in cucumber leaves, the seedlings of PS76, XTMC, and PWRG were cultivated under CK and HS conditions to detect sugar contents (Fig. 2A). After 15 days of treatment, there were no significant differences in the soluble sugar contents in PS76, XTMC, and PWRG under CK condition (Fig. 2B). However, HS induced the accumulation of soluble sugars in all three genotypes, with a significantly greater increase observed in the heat-sensitive cucumber PWRG than in the moderate heat-tolerant genotype XTMC and the heat-tolerant genotype PS76. Moreover, we measured glucose, fructose, and sucrose contents by high-performance liquid chromatography (HPLC). The contents of glucose, fructose, and sucrose were similar under CK condition among PS76, XTMC, and PWRG (Fig. 2C–E). In response to HS, XTMC and especially PWRG exhibited higher levels of glucose, fructose, and sucrose. In contrast, the contents of glucose, fructose, and sucrose in PS76 were barely increased under HS condition. Previous studies have confirmed that high sugar concentrations could induce stomatal closure [29, 30]. Based on these results, we supposed that the elevated sugar contents in XTMC and PWRG may promote the stomatal closure to prevent gas exchange. In comparison, the lower sugar contents in PS76 likely contributed to stomatal opening, facilitating leaf cooling and gas exchange in the leaves under HS condition. To verify the speculation, we investigated the role of extracellular sugar on stomatal aperture. Since PS76 contained relatively stable sugar content under HS condition, it was selected for the spraying experiments to investigate whether sugar application could regulate stomatal aperture. PS76 seedlings were treated with 50 mM mannitol (as an osmotic control), 50 mM glucose, 50 mM fructose, and 25 mM sucrose, respectively. Compared with mannitol, sugar treatments decreased stomatal aperture, suggesting that sugars played a non-osmotic role in regulating stomatal closure (Fig. 2F and G). To further explore the effect of sugar on heat resistance in cucumber, the seedlings of PS76 were grown under HS condition, and daily sprayed with different concentrations of glucose (Fig. S3A). After 15 days, we found the lower concentrations of glucose (20 and 40 mM) promoted the growth of seedlings, whereas higher concentrations (80 and 100 mM) inhibited growth and led to chlorophyll degradation and an increase in CAT activity (Fig. S3B–D).

### *Csiaglu* mutant plants were sensitive to long-term HS

**Figure 3:**
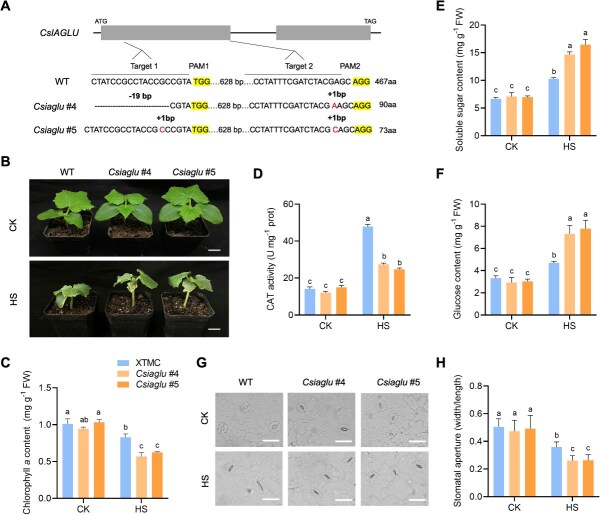
*Csiaglu* mutants were sensitive to HS. (A) Schematic diagram of edited sites in *Csiaglu* mutants. *Csiaglu* #4 mutant was 19-bp deletions in Target 1 and 1-bp insertion in Target 2, and the *Csiaglu* #5 mutant was 1-bp insertion in Target 1 and 1-bp insertion in Target 2. (B) Phenotypes of WT and *Csiaglu* mutants under CK and HS conditions. Scale bars, 2 cm. (C) Chlorophyll *a* content. (D) CAT activity. (E) Soluble sugar content. (F) Glucose content. (G, H) Stomatal response to HS in WT and *Csiaglu* mutants. Scale bars, 25 μm. Letters represent significant differences according to Tukey’s test (*P* < 0.05).

To investigate whether *CsIAGLU* contributed to the HS response, *Csiaglu* mutants were created by CRISPR-Cas9 technology in the wild-type (WT) control XTMC. *Csiaglu* #4 (19-bp deletion in Target 1 and 1-bp insertion in Target 2) and *Csiaglu* #5 (1-bp insertion in Target 1 and 1-bp insertion in Target 2) mutants were obtained, and both mutants generated a premature stop codon (Fig. 3A). WT and *Csiaglu* mutant lines were cultivated in the growth chambers under CK and HS conditions (Fig. 3B). Under HS condition, *Csiaglu* mutant leaves were more curled and yellowing than WT leaves. In response to HS, the Chl *a* content was decreased both in WT and *Csiaglu* mutant leaves, with much greater reduction in *Csiaglu* mutants (Fig. 3C). To examine whether *CsIAGLU* influenced the ROS scavenging system, we detected the activities of CAT in WT and *Csiaglu* mutants. The results indicated that CAT activity was rapidly increased in WT leaves compared with *Csiaglu* mutant leaves under HS condition (Fig. 3D). In addition, the heat tolerance values of WT (0.626) and *Csiaglu* mutants (0.331 and 0.318) were calculated by the equation in the evaluation system, which were consistent with their phenotypes. Moreover, the soluble sugar contents were significantly upregulated in *Csiaglu* mutant leaves with 2.07- and 2.37-fold higher, respectively, whereas it was only 1.54-fold higher in WT (Fig. 3E). Since the most abundant reversible IAA inactive forms are conjugates of IAA with sugars (such as glucose), we measured the glucose contents in WT and *Csiaglu* mutants [31, 32]. The contents of glucose in *Csiaglu* mutant leaves were significantly higher than WT under HS condition (Fig. 3F). We also observed the stomata of *Csiaglu* mutants and WT seedlings. As shown in Fig. 3G and H, *Csiaglu* mutants had a smaller stomatal aperture than that of WT seedlings. These data suggested that *Csiaglu* mutants were more sensitive to long-term HS with lower Chl content, stomatal aperture, and ROS scavenging activity, but higher sugar contents.

### CsDREB2C.L regulated the expression of *CsIAGLU* by directly binding to its promoter

**Figure 4:**
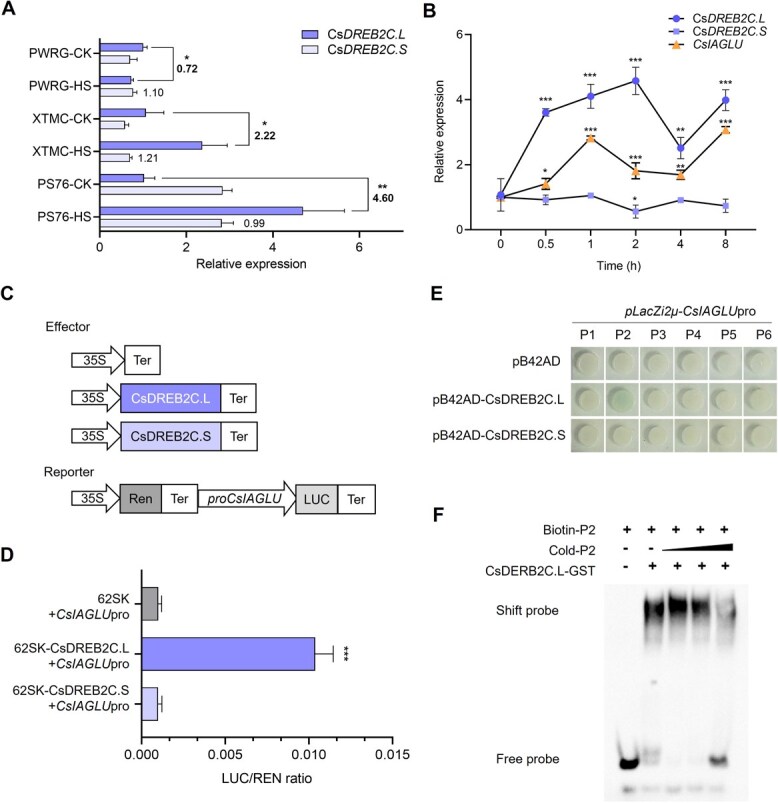
CsDREB2C.L positively regulates *CsIAGLU* expression. (A) Expression pattern of *CsDREB2C.L* and *CsDREB2C.S* under CK and HS conditions. The numbers represent the multiple changes of *CsDREB2C.L* and *CsDREB2C.S* in each genotype under HS condition related to CK condition. (B) Expression patterns of *CsDREB2C.L* and *CsDREB2C.S* in PS76 under HS. (C) Schematic diagrams of effectors and reporter. (D) The transient dual-luciferase assay of CsDREB2C.L/CsDREB2C.S. and *CsIAGLUpro*. (E) Y1H assay showed binding of CsDREB2C.L to the P2 region of *CsIAGLUpro*. (F) EMSAs showed that CsDREB2C.L bound to the P2 region of *CsIAGLUpro*. Asterisks represent significant differences determined by Tukey’s test. ^*^P < 0.05; ^**^P < 0.01; ^***^P < 0.001.

To further explore the potential regulatory mechanism of *CsIAGLU*, multiple *cis*-acting elements were identified in the promoter of *CsIAGLU* (Table S7). Notably, six adjacent DREB binding sites (−1740 to −1490 bp), an MYB-binding site, and a stress response element were identified in the *CsIAGLU* promoter region (Fig. S4A), suggesting that the expression level of *CsIAGLU* may be regulated by DREB under HS. Consistently, an HS-related DREB member, *CsDREB2C*, was found in the DEGs of XTMC under HS condition (Table S3). Cloning of *CsDREB2C* revealed two isoforms, namely *CsDREB2C.L* and *CsDREB2C.S* (Fig. S4B). Sequencing analysis showed that *CsDREB2C.L* transcript contained the canonical AP2-conserved domain and encoded a 372-amino acid (aa) protein, whereas the *CsDREB2C.S* transcript retained the first intron, leading to early termination and a truncated 38 aa protein. Next, we performed quantitative real-time polymerase chain reaction (qRT-PCR) using specific primers to examine the expression patterns of *CsDREB2C.L* and *CsDREB2C.S* in PWRG, XTMC, and PS76 under CK and HS conditions (Fig. 4A, Fig. S4C). As shown in Fig. 4A, *CsDREB2C.L* was downregulated in PWRG, while it was significantly upregulated in XTMC (*P* < 0.05) and PS76 (*P* < 0.01) under HS condition. The expressions of *CsDREB2C.S* were not induced under HS condition. Furthermore, we analyzed the expression levels of *CsDREB2C.L* and *CsDREB2C.S* under short-term HS (42°C) in PS76. The results showed that *CsDREB2C.L* was rapidly upregulated at 0.5 h and reached a peak at 2 h, whereas *CsDREB2C.S* expression was not induced in PS76 (Fig. 4B). These results indicated that *CsDREB2C.L* plays a crucial role in regulating HS resistance in cucumber.

To explore whether CsDREB2C.L or CsDREB2C.S could regulate *CsIAGLU* expression, we performed a dual-luciferase reporter assay in tobacco leaves. As shown in Fig. 4C and D, compared with the control group of 62SK, the activity of the *CsIAGLU* promoter was significantly increased in the presence of CsDREB2C.L, but not with CsDREB2C.S. Since the *CsIAGLU* promoter contains six DREB-binding elements (P1–P6, Fig. S4A), yeast one-hybrid (Y1H) assay was performed to confirm the binding sites. The results showed that CsDREB2C.L specifically bound to the P2 region of the *CsIAGLU* promoter (Fig. 4E). We conducted electrophoretic mobility shift assay (EMSA) to further investigate the binding site of the *CsIAGLU* promoter. The results showed that CsDREB2C.L specifically bound to DREB-binding elements in the P2 region (Fig. 4F). Additionally, qRT-PCR analysis showed that *CsIAGLU* exhibited a similar expression pattern to *CsDREB2C.L*, being strongly upregulated at 1 h under short-term HS in PS76 (Fig. 4B). This result implied that *CsDREB2C.L* may enhance HS resistance through stimulating *CsIAGLU* expression in cucumber.

### *CsDREB2C.L* and *CsIAGLU* positively regulated HS resistance

**Figure 5:**
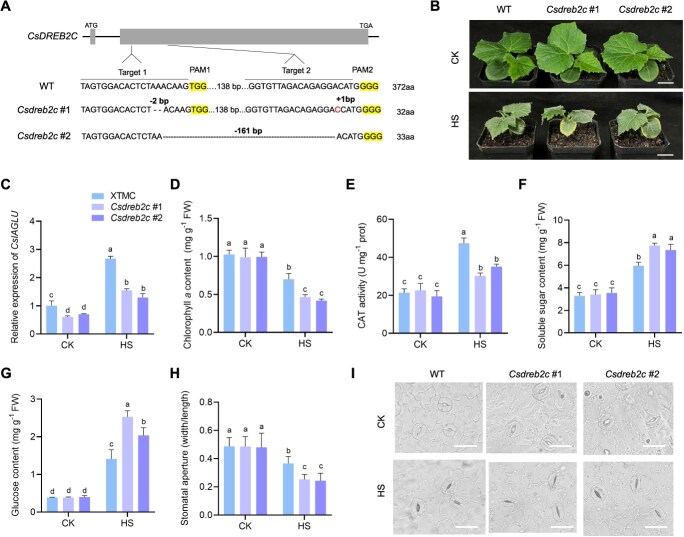
Heat tolerance analyses of *Csdreb2c* mutants. (A) The gene editing form of *Csdreb2c* mutants. *Csdreb2c* #1 mutant was 2-bp deletions in Target 1 and 1-bp insertion in Target 2, and the *Csdreb2*c #2 mutant was 161-bp deletions between Target 1 and Target 2. (B) Phenotypes of WT and *Csdreb2c* mutants under CK and HS conditions. Scale bars, 2 cm. (C) Expression analysis of *CsIAGLU* in WT and *Csdreb2c* mutants. (D) Chlorophyll *a* content. (E) CAT activity. (F) Soluble sugar content. (G) Glucose content. (H, I) Stomatal response to HS in WT and *Csdreb2c* mutants. Scale bars, 25 μm. Letters represent significant differences according to Tukey’s test (*P* < 0.05).

To investigate the role of *CsDREB2C.L* in regulating HS resistance, we generated gene-edited lines of *CsDREB2C* in XTMC and generated two independent transgenic lines. *Csdreb2c* #1 (with 2-bp deletion in Target 1 and 1-bp insertion in Target 2) and *Csdreb2c* #2 (with 161-bp deletion between Target 1and Target 2) were obtained (Fig. 5A). Both mutant lines resulted in premature termination proteins with 32 and 33 amino acids in length. The WT and *Csdreb2c* mutants were subjected to long-term heat treatment (Fig. 5B). The expression level of *CsIAGLU* was significantly downregulated in *Csdreb2* mutant compared with XTMC, indicating that CsDREB2C.L positively regulated the expression of *CsIAGLU* under HS condition (Fig. 5C). Phenotypic characterization showed that both mutant lines exhibited dehydrated and yellowing leaves under HS condition (Fig. 5B). Next, we detected Chl *a* content and CAT activity in WT and *Csdreb2c* mutant lines under CK and HS conditions. The results showed that both Chl *a* content and CAT activity were higher in WT seedlings compared to the *Csdreb2c* mutant lines (Fig. 5D and E). In contrast, *Csdreb2c* mutant seedlings accumulated higher levels of soluble sugars and glucose (Fig. 5F and G). Under HS condition, the stomatal apertures were respectively decreased by 47.8% and 49.4% in *Csdreb2c* mutant lines, while it was reduced by 25.1% in WT (Fig. 5H and I).

**Figure 6:**
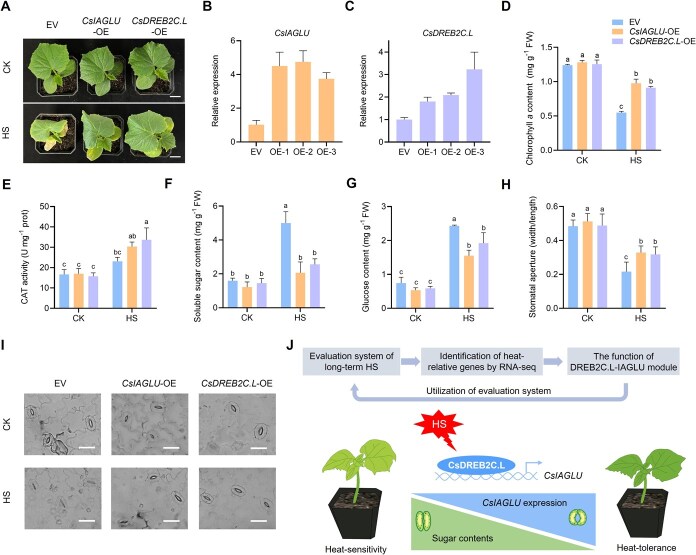
*CsIAGLU* and *CsDREB2C.L* positively regulate HS resistance. (A) Phenotypes of EV, OE-*CsIAGLU*, and OE-*CsDREB2C.L* seedlings under CK and HS conditions. (B) Expression analysis of *CsIAGLU* in EV and OE-*CsIAGLU* seedlings. (C) Expression analysis of *CsDREB2C.L* in EV and OE-*CsDREB2C.L* seedlings. (D) Chlorophyll *a* content. (E) CAT activity. (F) Soluble sugar content. (G) Glucose content. (H, I) Stomatal response to HS in EV, OE-*CsIAGLU*, and OE-*CsDREB2C.L* seedlings. Scale bars, 25 μm. (J) Proposed model of the CsDREB2C.L-CsIAGLU module under HS. Letters represent significant differences according to Tukey’s test (*P* < 0.05).

To further verify the function of *CsIAGLU* and *CsDREB2C.L* in HS resistance, both *CsIAGLU* and *CsDREB2C.L* were overexpressed in cucumber cotyledons, which were then subjected to HS condition for 4 days (Fig. 6A–C). Compared with the empty vector line (EV), *CsIAGLU*-OE and *CsDREB2C.L*-OE lines exhibited greater tolerance to HS. Degreening and wilting of cotyledons caused by HS were more obvious in EV seedlings than in *CsIAGLU*-OE and *CsDREB2C.L*-OE seedlings. Compared with EV seedlings, *CsIAGLU*-OE and *CsDREB2C.L*-OE lines exhibited higher Chl *a* contents and CAT activities under HS condition (Fig. 6D and E). We also measured the contents of soluble sugar and glucose in EV, *CsIAGLU*-OE, and *CsDREB2C.L*-OE seedlings. The soluble sugar contents were respectively increased by 1.70- and 1.75-fold in *CsIAGLU*-OE and *CsDREB2C.L*-OE seedlings, whereas it was significantly upregulated in EV seedlings with 3.13-fold (Fig. 6F). In response to HS, EV seedlings accumulated more glucose, whereas *CsDREB2C.L*-OE and *CsIAGLU*-OE seedlings showed lower glucose levels (Fig. 6G). In addition, stomatal apertures were significantly larger in *CsDREB2C.L*-OE and *CsIAGLU*-OE seedlings compared to EV seedlings (Fig. 6H and I). These results indicated that *CsDREB2C.L* and *CsIAGLU* acted as positive regulators of HS resistance in cucumber.

## Discussion

Cucumber, as a critical Cucurbitaceae crop cultivated throughout the world, is sensitive to environmental stress, especially HS. HS severely affects the photosynthesis and transport capacity of cucumber plants, ultimately leading to a decline in yield and quality. Along with climate warming, it is essential to establish an evaluation system and explore the molecular mechanism of long-term heat tolerance at the cucumber seedling stage. In this study, we established a long-term heat tolerance evaluation system, in which Chl *a* and CAT were selected as representative indicators of the photosynthetic system and antioxidant system, respectively.

In cucumber, some heat-related QTLs and candidate genes have been identified at the two-leaf stage and adult stage through QTL mapping and genome-wide association mapping [21, 24, 33]. However, the regulatory mechanisms underlying these findings have not been explored. Here, we found that sugar metabolism plays a vital function in heat response. Previous studies have shown that sugars not only provide carbon sources but also act as signal molecules in the regulation of plant growth, development, secondary metabolism, and response to biotic and abiotic stress. Among these pathways, hexokinase (HXK), SNF1-related protein kinases 1 (SnRK1), and target of rapamycin (TOR) are key components of sugar signaling [34–36]. In cucumber, sugar signaling has been reported to participate in fertility and fruit development via regulating metabolic enzymes and sugar transporters [37, 38]. We observed that the heat-tolerant genotype PS76 contained relatively stable sugar contents under HS condition, while the heat-sensitive genotype PWRG accumulated higher sugar contents (Fig. 2). Furthermore, increased temperature impacts stomatal conductance and response, and tolerant crop increases stomatal density or movement to improve gas exchange under HS [13, 39]. Therefore, we speculated that HS regulated stomatal movements via modulating sugar metabolism. In this study, we observed that the addition of sugars induced stomatal closure in PS76 seedlings. This finding is consistent with previous studies in *Arabidopsis*, which showed that sugars can promote stomatal closure [29]. In addition, the continuous application of glucose affected the heat tolerance of cucumber seedlings: low concentrations of glucose promoted the growth of seedlings, but high concentrations of glucose decreased the heat tolerance of seedlings (Fig. S3). A recent study revealed that low concentrations of sucrose induced stomatal opening under red light, while high concentrations of sucrose depressed stomatal conductance, implying the guard-cell was regulated by sugar in a concentration-dependent manner [30].

Stress-induced leaf senescence is accompanied by the accumulation of sugars, namely ‘plant diabetes’ [40, 41]. Exogenous sucrose treatment could decrease the Chl content and improve the electrolyte leakage with senescence symptoms in detached leaves of tobacco [42]. In wheat (*Triticum aestivum*), the sugar transport protein TaSTP3 enhanced the susceptibility to biotrophic fungi in leaves with higher sucrose content [43]. Similarly, we found that the *Csiaglu* mutant seedlings caused stomatal closure and chlorophyll degradation due to sugar accumulation, which eventually led to a sensitive response to long-term HS (Fig. 3). Previous studies have demonstrated that UGTs contributed to stress tolerance by regulating secondary metabolites under the control of TFs [44, 45]. In this study, we identified CsDREB2C as a transcriptional regulator of *CsIAGLU*, which was previously reported to interact with CsIVP in regulating HS response [46]. In *Arabidopsis*, AtDREB2C upregulated the expression of *AtHsfA3* in response to HS [47]. *CsIAGLU* expression was specifically activated by the canonical CsDREB2C.L. Notably, *Csdreb2c* mutant seedlings were more sensitive to long-term HS with higher soluble sugar content, but lower Chl *a* content and CAT activity. On the contrary, overexpression of *CsIAGLU* and *CsDREB2C.L* could reduce sugar accumulation, promote stomatal opening, and maintain Chl *a* content, thereby enhancing heat resistance. Furthermore, sequence alignment revealed that IAGLU and DREB2C were highly conserved among several cucurbit species, including melon, watermelon, wax gourd, and zucchini, implying that the DREB2C-IAGLU module may function similarly across these species (Fig. S5). These findings provide a valuable strategy for enhancing heat tolerance by upregulating the expression levels of *CsDREB2C.L* and *CsIAGLU* during cucumber breeding.

In summary, our findings reveal a novel regulatory module involving CsDREB2C.L and CsIAGLU that contributes to HS resistance in cucumber. Under HS, *CsDREB2C.L* is rapidly and strongly induced, particularly in the heat-tolerant genotype PS76. CsDREB2C.L can directly bind to the promoter of *CsIAGLU*, activating its expression. Both *CsDREB2C.L* and *CsIAGLU* were shown to positively regulate heat resistance by reducing soluble sugar accumulation, maintaining higher Chl *a* content and CAT activity, and promoting stomatal opening. Loss-of-function mutants of *CsDREB2C* or *CsIAGLU* exhibited increased sugar accumulation, premature stomatal closure, and more severe chlorophyll degradation under HS condition. These results suggest that the CsDREB2C.L-CsIAGLU module may enhance sugar metabolism homeostasis and facilitate stomatal opening, ultimately improving the plant’s capacity to tolerate prolonged HS. This mechanistic insight offers valuable targets for genetic improvement of thermotolerance in cucurbit crops (Fig. 6J).

## Materials and methods

### Plant material and experimental conditions

A total of 10 cucumber (*C. sativus* L.) genotypes were selected to establish the evaluation system of long-term heat tolerance, including four Southern China-type cucumbers (CU2, 32X, GFC, and PWRG), three Northern China-type genotypes (9930, XTMC, and 461), and three America cucumbers (PS76, AM218, and Gy14). The cucumber genotypes were cultivated in the growth chambers with 70%–80% relative humidity and light intensity at 300 μ mol m^−2^ s^−1^ (AS-R850, Xunon instrument, China) at the Sanya Institute of China Agricultural University (Hainan, China). Seeds were imbibed in sterile water and transplanted into plastic pots at 26°C. Upon germination, seedlings were transplanted to CK and HS conditions for long-term (15 days, the commonly used period for transplanting) treatment. Seedlings were cultivated under a 16/8 h day/night photoperiod and 26°C/22°C day/night temperature as CK condition, and under 42°C/28°C day/night temperature as HS condition. Seedlings were watered quantitatively every 2 days to ensure adequate moisture.

### Physiological measurement

The hypocotyl lengths of seedlings were measured after 15 days of CK and HS treatments. The HII was determined with five grades using a modified protocol as previously described [24]:

Class 0: no damage symptoms; Class 1: edges of the cotyledons and true leaf were dehydrated and yellowing; Class 2: less than one-third of the cotyledons and true leaf area was dehydrated and yellowing; Class 3: less than one-half of the cotyledons and true leaf area was dehydrated and yellowing; Class 4: more than one-half of the cotyledons and true leaf area was dehydrated and yellowing.

HII was calculated by the formula as follows:


\begin{equation*} \textrm{HII}=\sum \left(i\times Ni\right)/N\times 4\times 100. \end{equation*}

$i$ represented the heat injury grade, and $Ni$ represented the number of seedlings of different grades, and *N* indicated the total number of seedlings.

To detect the chlorophyll and carotenoid contents, leaf samples were ground and immersed in 95% ethyl alcohol for 2 days at room temperature in darkness. Absorbances of liquid supernatants were determined at A_470_, A_649_, and A_665_, and the contents were calculated as previously described [48]. The SOD, POD, and CAT activities were detected via spectrophotometry using the corresponding Assay Kit (Suzhou Keming Biotechnology Institute, China). To examine the germination rate of each genotype, the seeds were imbibed and germinated in sterile water at 26°C and 42°C, respectively. The germination rate was measured after 2 days. In each experiment, at least three technique replications and three biological replicates were performed.

### Evaluation system for cucumber seedling in response to HS

Principal component analysis was performed with the heat resistance factor of physiological data, and three principal components were extracted. The original data were transformed to the heat resistance factor (*h*) according to the equation:

*h*  $=$ Measured value under HS condition/Measured value under CK condition $\times 100$%.

Heat resistance values were calculated as previously described using the equations as follows [7, 9]:

Membership function value (*Ui*) $=$ (X*_i_*  $-$ X_min_)/(X_max_  $-$ X_min_);

Weight of composite index (*Wi*) $=$ Pi$/\sum_{i=1}^n\mathrm{Pi}.$

Pi represents the contribution variance of *i^th^* composite indicator;

Heat tolerance value (H) $=\sum_{i=1}^n Ui\times Wi$

*i* denotes the number of principal components, such as 1, 2, . . ., *n*.

Principal component analysis, cluster analysis, and regression analysis were performed by IBM SPSS statistics software (version 22.0, SPSS Inc.). The cluster analysis was carried out according to the heat tolerance value (H) of the 10 genotypes. The regression equation was identified by stepwise regression analysis. In this analysis, the H acted as the dependent variable, and the heat resistance coefficient of each index acted as the independent variable.

### Transcriptomic analysis

The cucumber seedlings of XTMC were sown and cultivated in the growth chambers under CK and HS conditions, respectively. After 15 days, the samples were collected from six independent seedlings as one biological replicate with three biological replicates, and were transcriptome sequenced at Wuhan MetWare Biotechnology Co., Ltd. (Wuhan, China). The filtered clean reads were mapped to the reference genome of cucumber (Chinese Long_V3) using HISAT2 software. Feature Counts was used to calculate normalized gene expression as fragments per kilobase of transcript per million fragments mapped (FPKM) [49]. DEGs were detected by DESeq2 between different samples with |log_2_Fold change| ≥ 1 and a *P*-value < 0.05 [50]. GO and KEGG analyses were performed to annotate the information of DEGs [51, 52].

### Determination of sugar contents and stomatal conductance

The cucumber seedlings were cultivated in the growth chambers under CK and HS conditions as previously described. After 15 days, leaf samples were collected for the determination of soluble sugar contents. The contents of soluble sugar were measured by spectrophotometry using the corresponding Assay Kit (Suzhou Keming Biotechnology Institute, China). High-performance liquid chromatography was performed on ACQUITY UPLC H-Class system (Waters, MA, USA) to measure the contents of glucose, fructose, and sucrose.

The 10-day-old seedlings of PS76 were sprayed with 50 mM mannitol (as an osmotic control), 50 mM glucose, 50 mM fructose, and 25 mM sucrose. After 3 h, stomatal aperture was observed by an optical microscope (Olympus D72, Tokyo, Japan) and analyzed by ImageJ software. Each experiment was performed with three biological replicates.

### Cucumber transformation

The CRISPR-Cas9 system was used to generate the *Csiaglu* and *Csdreb2c* mutants in the cucumber genotype XTMC using cotyledon transformation method as previously described [27, 53]. Genomic DNA was extracted from transgenic plants to detect the mutant lines with specific primers. All primers used in this study were listed in Table S8.

*CsIAGLU* or *CsDREB2C.L* was cloned into the pCAMBIA1300-GFP to generate 35S:*CsIAGLU* and 35S:*CsDREB2C.L* for overexpression. The recombinant vectors and empty vector were separately transformed into *Agrobacterium tumefaciens* strain EHA105 and transiently overexpressed in cucumber cotyledons as previously reported [54]. After 3 days postinfiltration, the seedlings were cultivated under HS condition for 4 days.

### Quantitative real-time PCR analysis

Total RNA extraction, reverse transcription, and qRT-PCR were performed by RNA extraction kit (Tiangen, Beijing, China), HiScript III All-in-one RT SuperMix Perfect for qPCR (Vazyme, Nanjing, China), and Taq Pro Universal SYBR qPCR Master Mix (Vazyme, Nanjing, China), respectively. The cucumber ubiquitin gene (*CsaV3_5G031430*) was used as the internal reference.

### Transient transcription dual-luciferase reporter assay

Approximately 2000-bp promoter regions of *CsIAGLU* were cloned into pGreen0800-LUC as a reporter, and the full-length coding sequence (CDS) of *CsDREB2C.L* and *CsDREB2C.S* were cloned into pGreen62SK vector as effectors. The reporter and effectors were respectively introduced into *A. tumefaciens* strain GV3101 (pSpoup-p19), and infiltrated into young *Nicotiana benthamiana* leaves. The firefly LUC and Renilla LUC were detected after 3 days of incubation by the dual-Luc Assay System (Promega).

### Yeast one-hybrid assays

The full-length CDS of *CsDREB2C.L* and *CsDREB2C.S* were cloned into pB42AD vector as effectors. Six predicted binding elements of DREB transcriptional factor were screened in the promoter of *CsIAGLU*, and the sequences around the elements were repeated three times and were cloned into the pLacZi2μ vector, respectively, as reporters. The effectors and reporters were cotransformed into *Saccharomyces cerevisiae* strain EGY48, and examined on SD/-Trp/-Ura and SD/-Trp/-Ura/X-Gal plates (Coolaber, China) for blue color development.

### Electrophoretic mobility shift assay

The CDS of *CsDREB2C.L* was cloned into the pET-28a vector and transformed into *Escherichia coli* strain BL21. The core-binding element of *CsIAGLU*pro was synthesized and labeled with biotin at the 5′ end. EMSA was performed using a Light Shift Chemiluminescent EMSA Kit (Biyuntian, China).

## Supplementary Material

Web_Material_uhaf341

## Data Availability

The data that support the findings of this study are included in the manuscript or supplementary materials. The RNA-seq data have been deposited in the China National Center for Bioinformation National Genomics Data Center (https://ngdc.cncb.ac.cn/) under accession number CRA025585.
